# Supplementary material for: Hybrid Models and Biological Model Reduction with PyDSTool
Source: PLoS Comput Biol. 2012 Aug 9;8(8):e1002628. doi: 10.1371/journal.pcbi.1002628 (PMC3415397; doi:10.1371/journal.pcbi.1002628)
Supplement: Text S4 — Complete source code for the PyDSTool package (version 0.88.120504). Includes API documentation and help files linking to web pages. This file is identical to the current public release on Sourceforge.net. (ZIP) [file pcbi.1002628.s004.zip › PyDSTool/html/PyDSTool.Toolbox.optimizers.optimizer.standard_optimizer_modifying-pysrc.html]

xml version="1.0" encoding="ascii"?


PyDSTool.Toolbox.optimizers.optimizer.standard\_optimizer\_modifying


| Home | Trees | Indices | Help | PyDSTool |
| --- | --- | --- | --- | --- |

Package PyDSTool ::
Package Toolbox ::
Package optimizers ::
Package optimizer ::
Module standard\_optimizer\_modifying

[hide private]

[frames] | no frames]

# Source Code for Module PyDSTool.Toolbox.optimizers.optimizer.standard\_optimizer\_modifying

```
 1   
 2  # Matthieu Brucher 
 3  # Last Change : 2007-08-10 23:10 
 4   
 5  """ 
 6  A standard optimizer with a special object that modifies the resulting set of parameters 
 7  """ 
 8   
 9  import optimizer 
10   


11 -class StandardOptimizerModifying(optimizer.Optimizer):


12    """ 
13    A standard optimizer, takes a step and finds the best candidate 
14    Must give in self.optimalPoint the optimal point after optimization 
15    After each iteration the resulting optimization point is modified by a call to a function 
16    """ 


17 -  def __init__(self, **kwargs):


18      """ 
19      Needs to have : 
20        - an object function to optimize (function), alternatively a function ('fun'), gradient ('gradient'), ... 
21        - a way to get a new point, that is a step (step) 
22        - a criterion to stop the optimization (criterion) 
23        - a starting point (x0) 
24        - a way to find the best point on a line (line_search) 
25      Can have : 
26        - a step modifier, a factor to modulate the step (step_size = 1.) 
27        - a pre-modifier, that acts on the set of parameters before an iteration (pre_modifier) 
28        - a post-modifier, that acts on the set of parameters after an iteration (post_modifier) 
29      """ 
30      optimizer.Optimizer.__init__(self, **kwargs) 
31      self.stepKind = kwargs['step'] 
32      self.optimalPoint = kwargs['x0'] 
33      self.lineSearch = kwargs['line_search'] 
34      self.pre_modifier = kwargs.get('pre_modifier') 
35      self.post_modifier = kwargs.get('post_modifier') 
36   
37      self.state['new_parameters'] = self.optimalPoint 
38      self.state['new_value'] = self.function(self.optimalPoint) 
39   
40      self.recordHistory(**self.state)

41   


42 -  def iterate(self):


43      """ 
44      Implementation of the optimization. Does one iteration 
45      """ 
46      self.state['unmodified_old_parameters'] = self.optimalPoint 
47      self.state['unmodified_old_value'] = self.state['new_value'] 
48   
49      if self.pre_modifier: 
50        self.optimalPoint = self.pre_modifier(self.optimalPoint) 
51      self.state['old_parameters'] = self.optimalPoint 
52      self.state['old_value'] = self.function(self.optimalPoint) 
53   
54      direction = self.stepKind(self.function, self.optimalPoint, state = self.state) 
55   
56      self.optimalPoint = self.lineSearch(origin = self.optimalPoint, function = self.function, state = self.state) 
57   
58      self.state['unmodified_new_parameters'] = self.optimalPoint 
59      self.state['unmodified_new_value'] = self.function(self.optimalPoint) 
60   
61      if self.post_modifier: 
62        self.optimalPoint = self.post_modifier(self.optimalPoint) 
63      self.state['new_parameters'] = self.optimalPoint 
64      self.state['new_value'] = self.function(self.optimalPoint) 
65   
66      self.recordHistory(**self.state)

67
```

  


| Home | Trees | Indices | Help | PyDSTool |
| --- | --- | --- | --- | --- |

|  |  |
| --- | --- |
| Generated by Epydoc 3.0.1 on Fri May 4 15:24:25 2012 | http://epydoc.sourceforge.net |
